# Isolated Penile Edema After Diagnostic Paracentesis

**DOI:** 10.7759/cureus.7329

**Published:** 2020-03-19

**Authors:** Israel Ojalvo, Andrew Salib, Carlos Rodriguez

**Affiliations:** 1 Medicine, Sidney Kimmel Medical College, Thomas Jefferson University Hospital, Philadelphia, USA; 2 Urology, Thomas Jefferson University Hospital, Philadelphia, USA; 3 Emergency Medicine, Thomas Jefferson University Hospital, Philadelphia, USA

**Keywords:** paracentesis, genital edema, penile edema, emergency medicine, liver disease, urology, complications

## Abstract

Diagnostic paracentesis is a routinely practiced, typically safe procedure performed in the emergency department. Genital swelling post-paracentesis is a rare complication with few documented case reports. We report a case of isolated penile edema after a diagnostic paracentesis performed in the emergency department. The patient is a 63-year-old male who came to the emergency department with a two-day history of isolated penile swelling after undergoing a diagnostic paracentesis in the emergency department as part of his workup during a recent hospital admission. On exam, the paracentesis site was noticeably low, beneath the inguinal ligament on the right side. His genital exam showed a circumcised penis with significant soft tissue swelling that involved the entire penile shaft sparing the glans and scrotum. There was no penile tenderness on palpation or urethral discharge. The testicles and scrotum revealed no signs of edema or tenderness, hernias, or abnormal lie. Of note, the patient reported that he had a less severe episode of penile swelling approximately one year ago after a paracentesis in a similarly low site, which resolved spontaneously. The features and timing of this presentation, added to the patient’s previous episode over a year ago, pointed to this being a sequela of the paracentesis he had undergone during his last hospital stay. After evaluation and consultation with the urology service, he was discharged home with expectant management and outpatient follow-up. His symptoms resolved spontaneously after one week. To our knowledge, there have been no published reports of isolated penile edema after a diagnostic paracentesis. This case could be used when teaching the proper technique for performing a paracentesis and its potential complications.

## Introduction

Patients who present to the emergency department (ED) with new-onset ascites, patients with worsening ascites in the setting of liver disease, or those with clinical deterioration may require a diagnostic or therapeutic paracentesis [1,2]. Paracentesis is seen as a typically safe procedure that can be performed under ultrasound guidance to relieve abdominal distension and provide a specimen for analysis and culture [3-5]. Of the rare complications post-paracentesis, the best chronicled are ascitic fluid leak (most common), bleeding, bowel perforation, and infection [5,6].

Genital swelling post-paracentesis is a rare complication with few documented case reports. We performed a literature review using PubMed, Google Scholar, and Scopus, which showed that there has never been a reported case on isolated penile edema post-paracentesis. Our search keywords included paracentesis, genital edema, scrotal edema, and penile edema. Our literature search yielded only three case reports of swollen genitalia after a paracentesis dating back to the 1970s, with two of them describing post-paracentesis scrotal edema and one case of labial edema [7-10]. Here we report a case of isolated penile edema after a diagnostic paracentesis performed in the ED. Written consent was obtained from the patient for the writing and use of images in this case report.

## Case presentation

This is a case of a 63-year-old male who presented to the ED with a two-day history of painless penile shaft swelling. He had a past medical history of liver cirrhosis, hepatitis C, and alcohol abuse. The patient stated that the swelling began one day prior to presentation and that it was not improved or worsened by any specific factors, was painless, and was not associated with other symptoms such as difficulty urinating, dysuria, testicular pain or swelling, or nausea. He also denied penile or groin trauma, or being sexually active. 

**Figure 1:**
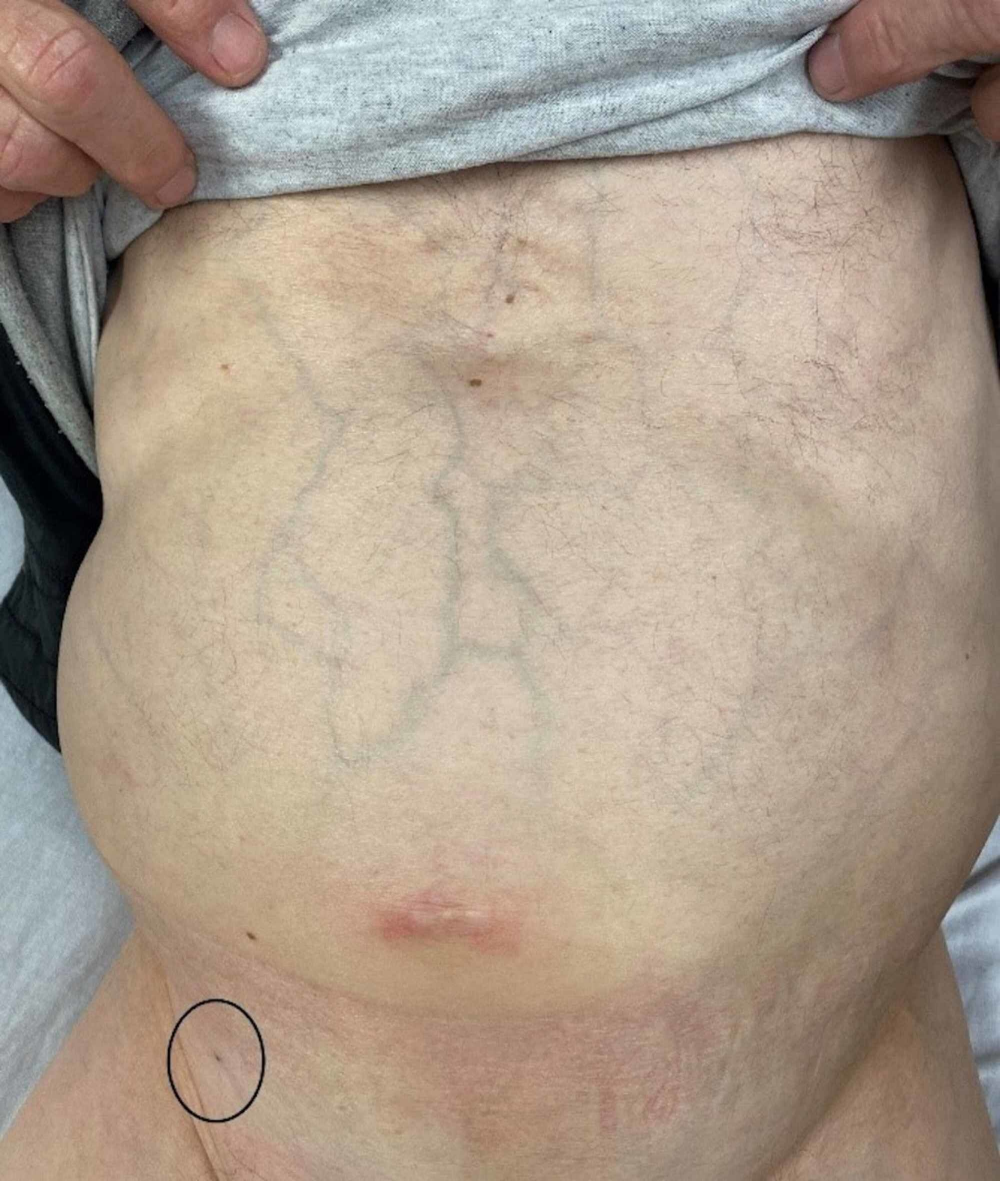
The paracentesis site from three days prior (circle)

On physical exam, the patient was well appearing and in no apparent distress. His abdomen was slightly distended with pronounced abdominal veins but soft and without tenderness to palpation. His right inguinal area had a piece of gauze taped to his skin, and upon removal, we noticed a puncture wound which was clean and dry and was noted to be a few centimeters medial to the patient’s right anterior inferior iliac spine in the inguinal region and appeared to be below the inguinal ligament (Figure 1).

**Figure 2:**
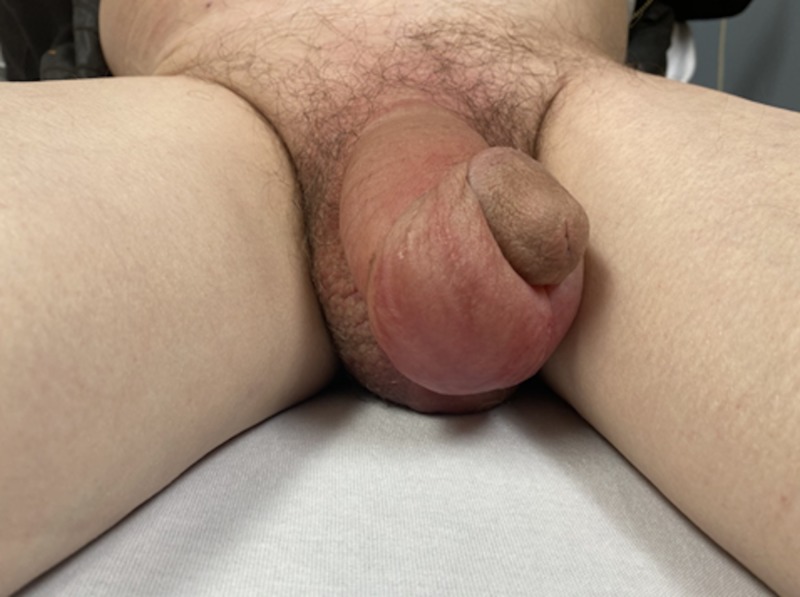
Notable edema of the penile shaft without scrotal swelling

The site was clean and dry and did not exhibit any swelling, tenderness, erythema, or warmth. His genital exam showed a circumcised penis with significant soft tissue swelling that involved the entire penile shaft sparing the glans and scrotum (Figure 2). There was no penile tenderness on palpation or penile discharge. The testicles and scrotum revealed no signs of edema or tenderness, hernias, or abnormal lie. 

During our interview, we discovered that three days prior to the presentation, the patient visited the same ED for evaluation of an episode of upper gastrointestinal bleeding (UGIB) in a setting of decompensated cirrhosis for which he was evaluated and subsequently admitted to the hospital. During his ED evaluation, he underwent a diagnostic paracentesis that ruled out spontaneous bacterial peritonitis. Documentation about the procedure did not specify if ultrasound guidance was used. He was admitted for the management of decompensated cirrhosis and UGIB. Of note, the patient reports that he went to the ED with a less severe episode of penile swelling, which occurred approximately one year ago after a paracentesis, which resolved spontaneously without extra care. 

The features (i.e., site of paracentesis) and timing of this presentation, added to the patient’s previous episode over a year ago, pointed to this being a sequela of the paracentesis he had undergone during his last hospital stay rather than an acute infectious process such as cellulitis or spontaneous bacterial peritonitis. 

While in the ED, the patient was evaluated by the urology service. After their evaluation, we agreed that the swelling was likely a complication due to the paracentesis and they only recommended that the patient wear tight underwear for scrotal support. Urine analysis performed in the ED was negative for urinary tract infection and was sent out for culture which did not show bacterial growth. The patient was discharged home with return parameters and reassurance that the swelling would resolve over the next couple of days. During a subsequent telephone encounter, the patient reported that the swelling fully resolved about one week after our evaluation.

## Discussion

Paracentesis is a routine procedure performed at the patients’ bedside to provide symptomatic relief and assist in diagnostic efforts. With improvements in imaging modalities (i.e., ultrasound guidance), procedures like paracentesis have become more ubiquitous with relatively low complications [4,5]. Even though ultrasound guidance is not always necessary, it reduces the incidence of complications and injury to nearby structures compared to paracentesis based solely on physical exam [6]. Operator’s comfort and experience with the use of ultrasound play a major role in the outcomes of the procedure. In this case report, we explore a rare complication of paracentesis, isolated penile edema, which could be associated with a negative psychological impact due to genital disfigurement and slow recovery. 

We believe that this episode of penile edema is directly related to the location of the paracentesis entry point. Currently, there is no consensus on the best location for needle entry during an abdominal paracentesis, and the ideal location may differ between patients [6]. However, in general the bilateral lower quadrants of the abdomen are considered safer than the midline due to the increased thickness of the abdominal wall at the midline and increased risk of hematoma. One study considers the left lower quadrant ideal as it is not as thick as the infraumbilical midline and does not risk perforating a distended cecum (i.e., post-lactulose administration) in the right lower quadrant [11]. Others recommend paracentesis site in the relatively avascular areas beneath the umbilicus on either side laterally with caution to avoid the bilateral epigastric vessels, previous scarring, or engorged veins [6,12]. There is no literature supporting paracentesis below the inguinal ligament. 

This patient’s penile swelling can be attributed to two different mechanisms: low puncture site and hypoalbuminemia (average albumin during visit 3.0 g/dL (normal range 3.2-4.9 g/dL)) . Examining the patient’s paracentesis site showed that the needlestick was relatively low in the right lower quadrant just below the inguinal ligament. We hypothesize that the low needlestick led to a post-procedure leak which dissected the abdominal wall fascia and extended down to the penis causing swelling. The penis is surrounded by superficial dartos fascia and deep Buck’s fascia, which surround the corporal bodies and are important for erectile function. The superficial dartos fascia is continuous with the scrotum and perineum Colles’ fascia and the abdominal wall Scarpa's fascia [13]. Theoretically, a low paracentesis stick could lead to fluid tracking along the abdominal Scarpa's fascia and track down to the penile dartos fascia causing separation of soft tissue and swelling. 

The second contributing factor in this patient is his low protein state secondary to chronic liver disease. The patient suffers from alcoholic liver cirrhosis leading to hypoalbuminemia and low oncotic pressure, which in turn leads to fluid accumulation in the extravascular space and dependent areas such as the penis and scrotum if proper paracentesis technique is not observed.

Interestingly, this was the patient’s second episode with penile complications post-paracentesis. The reproducibility of this patient’s complication further demonstrates the anatomical relationship between the fascial layers of the abdomen and genitalia and the risk of such complications after paracentesis. Penile swelling in this setting is a self-limiting phenomenon, which resolved with conservative management. Even though this disfigurement is not as life-threatening as some of the other complications after a paracentesis, it can be distressing to patients and negatively affect their quality of life. We hope that this case report will provide an example of this uncommon complication to help providers identify it and manage it.

## Conclusions

This is the first written case report of a patient experiencing isolated penile swelling after a diagnostic paracentesis performed in the ED. We hope that this case report can be a valuable example of a potential rare complication post paracentesis. This case could be used when teaching the proper technique for performing a paracentesis and its potential complications.

## References

[1] Borzio M, Salerno F, Piantoni L (2001). Bacterial infection in patients with advanced cirrhosis: a multicentre prospective study. Dig Liver Dis.

[2] Chinnock B, Afarian H, Minnigan H, Butler J, Hendey GW (2008). Physician clinical impression does not rule out spontaneous bacterial peritonitis in patients undergoing emergency department paracentesis. Ann Emerg Med.

[3] Ge PS, Runyon BA (2016). Treatment of patients with cirrhosis. N Engl J Med.

[4] Mallory A (1978). Complications of diagnostic paracentesis in patients with liver disease. JAMA.

[5] De Gottardi A, Thévenot T, Spahr L (2009). Risk of complications after abdominal paracentesis in cirrhotic patients: a prospective study. Clin Gastroenterol Hepatol.

[6] McGibbon A, Chen GI, Peltekian KM, van Zanten SV (2007). An evidence-based manual for abdominal paracentesis. Dig Dis Sci.

[7] Pereira W, Seeff LB (1971). Sudden scrotal edema:old pearls?. Ann Intern Med.

[8] Conn HO (1971). Sudden scrotal edema in cirrhosis: a postparacentesis syndrome. Ann Intern Med.

[9] Blumberg C, Villaverde C, Gardner R (2016). Postparacentesis genital edema. Am J Med.

[10] Marks JW, Weil F (1971). Conn’s sudden labial edema. Ann Intern Med.

[11] Sakai H, Sheer TA, Mendler MH, Runyon BA (2005). Choosing the location for non-image guided abdominal paracentesis. Liver Int.

[12] Runyon BA (1986). Paracentesis of ascitic fluid. Arch Intern Med.

[13] Angermeier KW (2006). Surgical anatomy of the penis. Operative Urology at the Cleveland Clinic.

